# The effect of the combination therapy with chlorophyllin, a glutathione transferase P1-1 inhibitor, and docetaxel on triple-negative breast cancer invasion and metastasis in vivo/in vitro

**DOI:** 10.1007/s00210-025-03929-y

**Published:** 2025-02-27

**Authors:** Ayse Burus, Mehmet Ozcan, Hande Canpinar, Ozlem Bozdemir, Naciye Dilara Zeybek, Yasemin Bayazit

**Affiliations:** 1 Department of Medical Biochemistry, Hacettepe University Faculty of Medicine, Ankara, Turkey; 2 Department of Medical Biochemistry, Zonguldak Bulent Ecevit University Faculty of Medicine, Zonguldak, Turkey; 3 Department of Basic Oncology, Hacettepe University Cancer Institute, Ankara, Turkey; 4 Department of Stem Cell Sciences, Hacettepe University Graduate School of Health Sciences, Ankara, Turkey; 5 Department of Histology and Embryology, Hacettepe University Faculty of Medicine, Ankara, Turkey

**Keywords:** Triple negative breast cancer, Metastasis, Chlorophyllin, Antioxidants, Glutathione S-transferase, Combination therapy

## Abstract

The expression of glutathione S-transferase P1 (GSTP1) enzyme increases in cancer cells, leading to anticancer drug resistance. The antioxidant chlorophyllin has an inhibitory effect on GSTP1. In this study, we investigated the effect of chlorophyllin and its combined administration with the chemotherapeutic agent docetaxel on metastatic processes. For this purpose, both the 4T1 triple-negative breast cancer cell line and metastatic animal model were used. The MTT, flow cytometry, and wound healing assays were used to investigate cell viability, cell cycle, and cell migration, respectively. Total gelatinase activity, GST activity, and glutathione levels in cell and liver tissue lysates measured by colorimetric methods. Micrometastases were evaluated histochemically in liver tissue sections. As a result, the coadministration of chlorophyllin and docetaxel significantly inhibited cell migration in vitro. There was a significant decrease in the total gelatinase activity in vivo. We found that only combined treatment reduced the micrometastatic lesions in the liver tissues, though this reduction was not statistically significant. In conclusion, the coadministration of chlorophyllin and docetaxel may have a potential role in controlling metastatic processes by suppressing cell migration, gelatinase activity, and micrometastasis formation in triple-negative breast cancers.

**Graphical Abstract:**

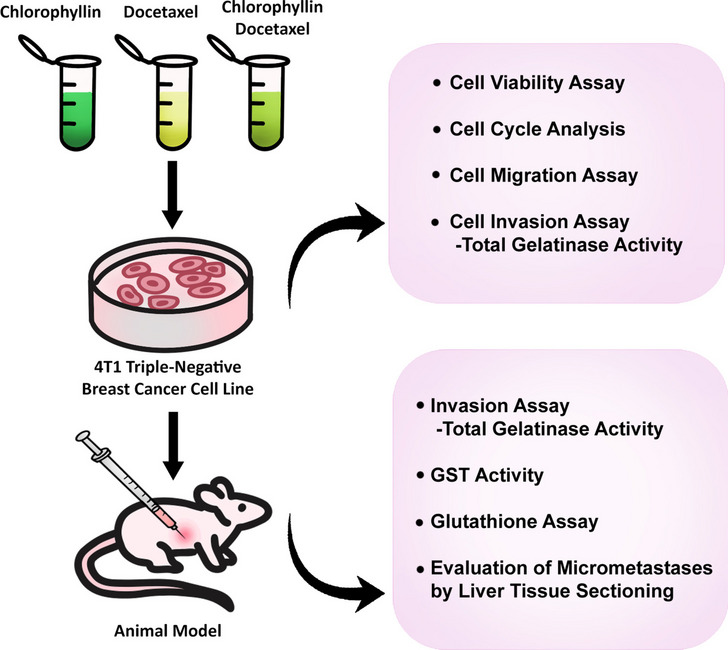

## Introduction

With an increasing incidence, breast cancer has the most common cancer type in women accompanied by high mortality rates (Braithwaite et al. 2016). In the treatment of breast cancer, different treatment options can be applied according to the subtypes of the disease. However, among these cancer subtypes, triple negative (ER^-^, PR^−^, and HER2^-^) breast cancer without receptor sensitivity is the most lethal subtype due to its heterogeneity, aggressiveness, and lack of treatment options (Nedeljkovic and Damjanovic 2019). Since there is no response to hormonal therapy or HER2-targeted drug therapies, chemotherapy remains one of the main treatment options, yet the prognosis remains poor due to high recurrence rates and drug resistance (Won and Spruck 2020, An et al. 2021).

One of the major challenges in TNBC treatment is the development of resistance to chemotherapeutic agents, which contributes to treatment failure and tumor progression. The glutathione S-transferase P1 (GSTP1) enzyme has been implicated in the mechanism of chemotherapy resistance by facilitating the detoxification of chemotherapeutic drugs, thereby reducing their intracellular accumulation and efficacy (Peter Guengerich et al. 2003, An et al. 2021).

In addition to drug resistance and recurrence of tumors, TNBC’s high metastatic potential, particularly its ability to invade distant tissues, remains a major cause of mortality (Wu et al. 2021). Metastasis is a multi-step biological process including the migration and invasion of cancer cells, and it requires the destruction of the extracellular matrix (ECM). It has been reported that matrix metalloprotease (MMP) enzymes are responsible for the proteolytic degradation of ECM, and they play a role in invasion and metastasis due to the high expression of these MMPs in various tumor tissues (Kleiner and Stetler-Stevenson 1999). Therefore, targeting MMP activity and metastatic pathways could improve TNBC treatment outcomes.

Docetaxel, a cytotoxic antimicrotubular agent, has been used in metastatic breast cancer treatment (Lyseng-Williamson and Fenton 2005). However, chemotherapy and radiotherapy can paradoxically induce resistance in a subset of cancer cells. This resistance is often associated with the upregulation of epithelial-mesenchymal transition (EMT), a process by which carcinoma cells acquire a mesenchymal-like phenotype, leading to increased invasiveness and metastatic potential (Rastegar-Pouyani et al. 2024). This presents a significant challenge in the treatment of TNBC, where limited treatment options and poor outcomes necessitate the development of new therapeutic strategies to enhance treatment efficacy and suppress metastatic progression.

Several studies reported that GSTP1 overexpression enhances cancer cell proliferation, invasion, and formation of metastatic nodules, while suppression of the enzyme has the opposite effect, which suggests GSTP1 acts as a positive regulator of metastatic processes (FeiFei et al. 2019). Accordingly, a study showed that GSTP1 could upregulate the STAT3 to promote distance tissue and lymphatic metastasis, indicating its regulatory role in signaling pathways related to metastatic processes (Wang et al. 2022). Current literature also highlights the eminent role of cancer stem cell population in tumors to promote metastatic processes (Steinbichler et al. 2020). Interestingly, increased GSTP1 expression has been shown to maintain the CSC phenotype in lung adenocarcinoma and facilitated metastasis through regulating EMT (Wang et al. 2023). In parallel, a study especially investigating the TNBC pathogenicity reported that GSTP1 is a critical metabolic driver in breast cancer cells undergoing EMT, with heightened aggressiveness and poor prognosis alongside increased proliferation and migration (Louie et al. 2016). Another study showed transfection of cells with miR-133b, a microRNA targeting GSTP1, downregulated certain MMP protein expression and reduced cell migration in non‑small cell lung cancer (Lin et al. 2018). All these findings suggest that GSTP1 overexpression, beyond its role in drug resistance, may contribute to the metastatic ability of cancers including promoting EMT, modulating MMPs, and upregulating certain signaling pathways, thus becoming an attractive target for metastasis research.

Plant-derived phytochemicals have become a considerable source in cancer therapy, especially since they have antitumor effects and are protective against the cytotoxic side effects of chemotherapeutic agents with their antioxidant properties (Choudhari et al. 2019, Rizeq et al. 2020). Chlorophyllin, a water-soluble salt derived from chlorophyll, has been known for its antioxidant properties and has shown anticarcinogen effects (Kumar et al. 2001). In one study, chlorophyllin has been shown to suppress fibrosarcoma cell migration and invasion with suppressing MMP secretion (Roomi et al. 2018). Moreover, in previous studies by our group, chlorophyllin has been shown to inhibit the GSTP1 enzyme, which demonstrates it may potentially increase the efficacy of chemotherapy by suppressing drug resistance (Musdal et al. 2013, Ozcan et al. 2018, Ozcan et al. 2021). Therefore, given the challenges posed by chemotherapy-induced EMT and the associated increase in invasiveness, the anti-migratory effects of chlorophyllin alongside GSTP1 inhibition could play a role in improving the outcomes of docetaxel therapy for metastatic TNBC.

Despite chlorophyllin’s therapeutic potential, its impact on metastasis, particularly in TNBC, remains largely unexplored. This study aims to fill this gap by evaluating the anti-metastatic activity of chlorophyllin, both alone and in combination with docetaxel, in the 4T1 breast cancer cell line and a mouse model created via inoculation of 4T1 cells in BALB/c. Therefore, we investigated the key processes associated with metastasis including migration, MMP-2 and MMP-9 (gelatinase) enzyme activity as well as the formation of micrometastases in liver tissue. Our results suggest that chlorophyllin may be a potential agent in metastatic breast cancer therapy and it needs further investigation.

## Methods

### Cell culture

The mouse triple-negative breast cancer cells, 4T1, were obtained from American Type Culture Collection (ATCC) and cultured in RPMI-1640 medium (Biowest, France) supplemented with L-glutamine (Biowest), 10% fetal bovine serum (FBS, Biowest), and 1% penicillin/streptomycin (Biowest) at 5% CO_2_ at 37 °C. All the experiments were performed with the cells that reached 80–90% confluency in T75 culture flasks.

### MTT assay

The number of viable cells was measured with the 3-(4,5-dimethylthiazol-2-yl)−2,5-diphenyltetrazolium bromide (MTT, Sigma‐Aldrich, USA) assay (Hansen et al. 1989). 4T1 cells (100 μL; 5 × 10^3^/well) were seeded into 96-well plates and incubated overnight at 37 °C. Varying concentrations (0, 12.5, 25, 50, 100, and 200 μM) of chlorophyllin (Sigma‐Aldrich) or docetaxel in 0.1% DMSO (Sigma‐Aldrich) were added to wells in triplicates and incubated for 24 or 48 h. The MTT solution (50 µL, 1 mg/mL) was added and incubated for 3 h at 37 °C, and then 100 µL isopropanol (Sigma‐Aldrich) was used to dissolve the formazan crystals. Further, optical densities were measured at 570 nm with a SpectraMax M2 microplate reader (Molecular Devices, USA). The cells incubated in a culture medium treated with 0.1% DMSO were used as controls.

### Cell cycle analysis

4T1 cells were treated with the IC50 concentrations of docetaxel (31.45 μM for 24 h and 34.4 μM for 48 h) or 50 μM chlorophyllin and incubated for 24 and 48 h. Then, trypsinized cells were washed and collected by centrifugation (1000 rpm, 5 min). After being fixed with 99% ethanol and overnight incubation, cells were washed and Rnase (Sigma-Aldrich), and propidium iodide (Sigma-Aldrich) was added to the cells. Following a 30-min incubation in the dark at room temperature, 5 × 10^4^ cells were counted and cell cycle analysis (ratio of G0/G1 and S + G2/M phases) was performed with a flow cytometer (Cytoflex; BeckmanCoulter) using dichotomous variable histograms.

### Wound healing assay

To investigate the effects of chlorophyllin, docetaxel, and their coadministration on cell migration in 4T1 cells in vitro, a standard wound healing assay was performed (Valster et al. 2005). Cells (5 × 10^4^) were seeded into 24-well plates and grown until 95–100% confluent. After the medium was removed, a linear wound was created by using a 200-μL pipette tip, and images were recorded at the baseline time point. Cells were washed with PBS (Sigma-Aldrich) and treated with chlorophyllin (50 μM), docetaxel (31.45 μM), and their combination in RPMI-1640 containing 2% FBS. Then, images of the wound surfaces were recorded after 24 h. Wound areas were measured using the wound healing size tool, an ImageJ software plugin (Suarez-Arnedo et al. 2020). Percent Motility Index was used to evaluate the migration ability of the cells.

### Establishment of animal model for metastasis

Animal studies were approved by the Hacettepe University Animal Experimentations Local Ethics Board (approval number: 2013/72‐10) before its commencement. Female BALB/c mice, aged 6–8 weeks, were purchased from Kobay Experimental Animal Laboratory (Ankara, Turkey) and housed at 21 °C temperature and 30–70% humidity under a 12-h light and darkness cycle and regular autoclave chow diet with water. The mammary tumor model was created by inoculating 5 × 10^4^ 4T1 cells into the mammary adipose tissue of 6–8 weeks old female Balb/c mice in physiological saline solution, and it was considered as day 0. On day 8, the mean tumor diameters reached 2 mm, and the mice were randomly divided into 4 groups for injections (*n* = 6). The control group received only 0.1 mL physiological saline solution in 1% DMSO; docetaxel group received 8 mg/kg docetaxel in 0.1 mL physiological saline solution with 1% DMSO; chlorophyllin group received 30 mg/kg chlorophyllin in 0.1 mL physiological saline solution with 1% DMSO, and chlorophyllin + docetaxel group received 8 mg/kg docetaxel + 30 mg/kg chlorophyllin in 0.1 mL physiological saline solution with 1% DMSO injections for each day throughout 10 days intraperitoneally. After the incubation period, mice were sacrificed. Extracted liver tissues were washed with cold physiological saline and stored at −80 °C. Further, liver tissues were processed for frozen sectioning, hematoxylin and eosin (H&E) staining, and then micrometastases were counted by examination of H&E-stained sections under the microscope.

### Gelatinase degradation assay

By using a gelatin breakdown assay kit (Abcam, Cambridge, UK), levels of gelatinase activity were assessed in both cell and liver tissue lysates. Cells were seeded in T75 culture flasks and treated with chlorophyllin (50 μM), docetaxel (31.45 μM), and their combination. The BCA method was used to calculate the protein concentration after preparing the cell and tissue lysate in accordance with the kit’s instructions (Shen 2019). Gelatinase assay buffer and 50 M FITC standard were produced in accordance with the kit’s instructions, and standards were then loaded to the 96-well plate in triplicate to draw the standard curve. Cell (10 μL) and 25-μL liver tissue sample lysates were added to each well, and gelatinase assay buffer was used to adjust the final volume to 50 μL/well. Then, 50 μL of substrate mix solution was added to the well of each sample, and positive control. The fluorescence was measured in kinetic mode at Ex/Em 490/520 nm for 2 h at 37 °C. Using the formula in the kit’ description, the levels of gelatinase activity were calculated and compared to the control.

### GST activity assay

Glutathione transferase (GST) activity in liver tissue samples was evaluated spectrophotometrically by measuring the initial rate of absorbance change at 340 nm, as described by Habig et al. (1974). First, homogenization buffer (100-mM potassium phosphate buffer, 1 mM EDTA, pH 7.0) was added to the liver samples (1:10 w/v). After the centrifugation at 10,000 ×g for 10 min, supernatants were obtained. Then, standard enzymatic assays were performed in 100-mM sodium phosphate buffer (containing 1 mM EDTA, pH 6.5), 1 mM GSH (Sigma-Aldrich), and 1 mM 1-chloro-2,4-dinitrobenzene (CDNB) (Sigma-Aldrich) at 30 °C. The molar absorption coefficient for CDNB was 5.3 mM^−1^·cm^−1^. Correction for the spontaneous nonenzymatic reaction was obtained by subtracting the reaction rate of GSH with CDNB in the absence of the enzyme from the rate in the presence of the enzyme.

### Glutathione assay

The liver tissue was homogenized in a cold solution of 100-mM phosphate buffer (with 1 mM EDTA, pH 7). In order to eliminate the precipitated protein, the supernatant was deproteinized using a 10% trichloroacetic acid (Sigma-Aldrich) solution and centrifuged at 10,000 g for 10 min at 4 °C. Glutathione was then analyzed in the supernatant. Using a kinetic assay, the amount of total glutathione was determined by continuously reducing 5,5′-dithiobis (2-nitrobenzoic acid) (Sigma-Aldrich) to 5-thio-2-nitrobenzoic acid in the presence of catalytic amount (nmol) of GSH (TNB), and GSH reductase and nicotinamide adenine dinucleotide phosphate (Sigma-Aldrich) regenerated the GSSG (oxidized GSH) that was produced. Then, using a spectrophotometric device (SpectraMax M2; Molecular Devices), the yellow product, TNB, was detected at 412 nm (Akerboom and Sies 1981).

### Histochemistry of tissue sections

Freeze-hardened liver tissue samples were cut into 5-µm thick sections at −20 °C by using a cryostat microtome (Leica CM1900 UV, IL, USA) and mounted on glass slides. Then, the sections were stained with hematoxylin and eosin (H&E). Twenty sections per tissue sample were taken and imaged with a camera (Leica DFC7000T) by using LAS X software. To quantify liver metastasis, the number of micrometastases (clusters of > 4 tumor cells) was counted and normalized to the total area covered during quantification.

### Statistical analysis

Statistical analysis of data was performed using the GraphPad Prism 8.3 software. Data are presented as *mean* ± *SD*, and analyzed by the Kruskal-Wallis test to determine the significance of non-parametric independent multiple groups. If *p*-values ≤ 0.05, the difference between groups was considered to be statistically significant.

## Results

### Cell viability

**Fig. 1:**
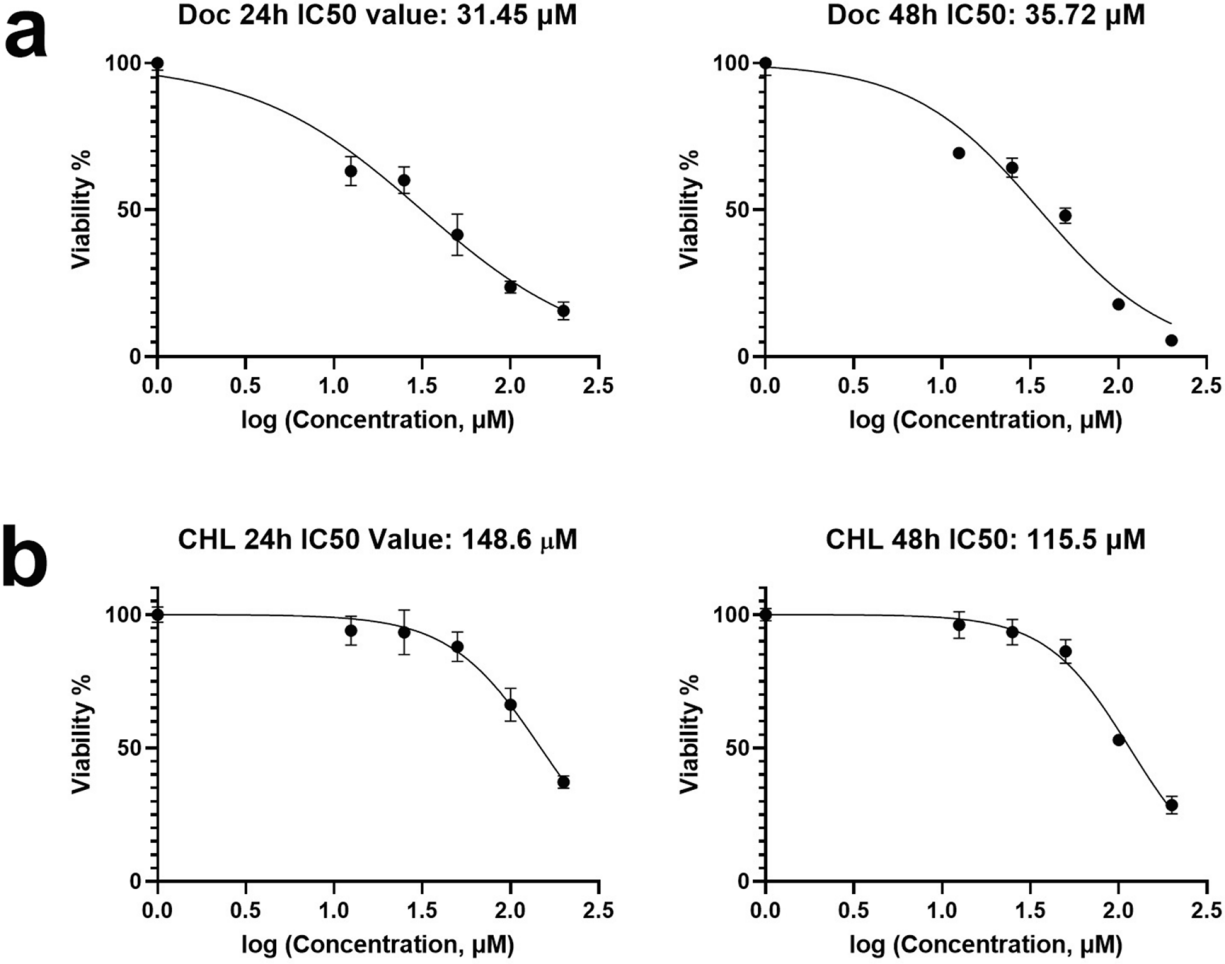
The MTT Assay. 4T1 cells were treated with 0, 12.5, 25, 50, 100, and 200 μM docetaxel (**a**) and chlorophyllin (**b**) for 24 and 48 h. The percentage of cell viability was calculated as the following formula: (viable cells) % = (OD of drug-treated sample/OD of the untreated sample) ×100. Doc, docetaxel; CHL, chlorophyllin. *Mean* ± *SD*. *n* = 3

Effects of docetaxel and chlorophyllin on the viability of 4T1 cells were examined using the MTT assay. The IC_50_ values for docetaxel were 31.45 ± 1.61 for 24 h, and 35.72 ± 1.68 for 48-h treatment (Fig. 1a), suggesting a dose-dependent inhibition of growth, while chlorophyllin has no high toxic effect on 4T1 cells (Fig. 1b) with the IC_50_ values was 148.6 ± 5.69 for 24 h, and 115.5 ± 2.98 μM for 48-h treatment.

### Cell cycle analysis

**Fig. 2:**
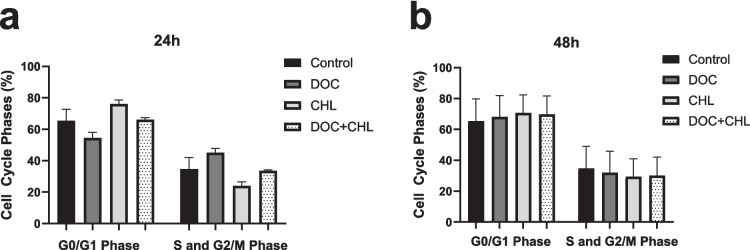
The percentage of 4T1 cells in different cell cycle phases after treatments with docetaxel, chlorophyllin, and their combination for 24 h (**a**) and 48 h (**b**). DOC, docetaxel; CHL, chlorophyllin. *Mean* ± *SD*. *n* = 3

After incubation with docetaxel (31.45 for 24 h and 35.72 μM for 48 h), chlorophyllin (50 μM for 24 and 48 h), and coincubation of both compounds, the distributions of cell cycle phases were analyzed by FCM. We found that while the docetaxel decreased the G0/G1 phase, both chlorophyllin and its coadministration with docetaxel led to a slight increase in the G0/G1 phase compared to the control group after 24 h of incubation; however, these changes were not statistically significant (Fig. 2a). Treatment groups showed a similar pattern to the control group within 48 h (Fig. 2b).

### Cell migration analysis

**Fig. 3:**
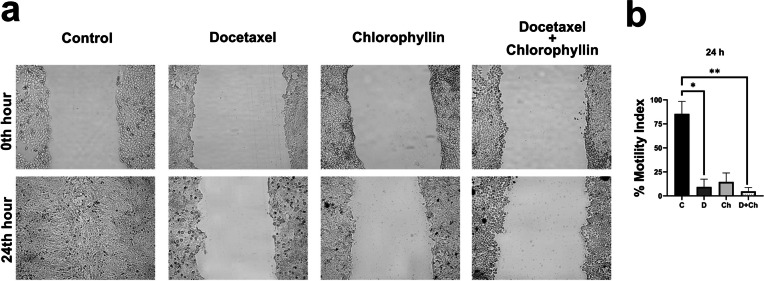
The images (**a**) of cell migration assay and the % Motility Indexes of 24 h (**b**). C, control; D, docetaxel; Ch, chlorophyllin. * *p* < 0.05, ** *p* < 0,01. *Mean* ± *SD*. *n* = 3

4T1 cells were treated with docetaxel (31.45 μM), chlorophyllin (50 μM), and both docetaxel and chlorophyllin for 24 h. The images of the result of the migration assay are represented in Fig. 3a. The results indicated that docetaxel blocked cell migration significantly, but this effect was higher when docetaxel was treated with chlorophyllin (Fig. 3b).

### Evaluation of total gelatinase activity

**Fig. 4:**
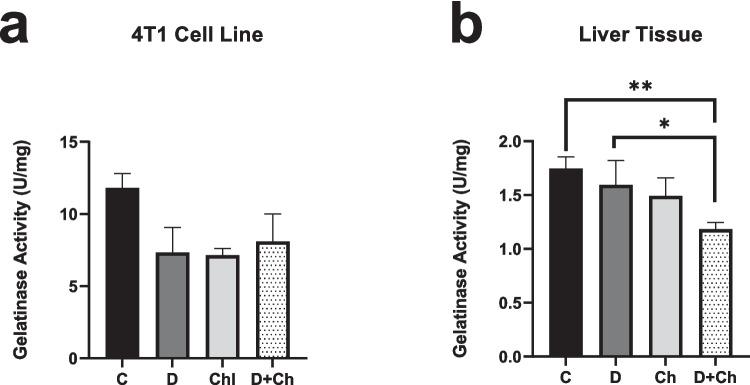
Gelatinase activity in 4T1 cell line (**a**) and liver tissue (**b**). C, control; D, docetaxel; Ch, chlorophyllin. * *p* < 0.05, ** *p* < 0.01. *Mean* ± *SD*

The gelatinase activity in both 4T1 cells and liver tissue samples was analyzed by using a gelatin degradation assay. The results of the gelatin degradation assay showed that the decrease in gelatinase activity was not significant in the 4T1 cell line; however, the coadministration of chlorophyllin and docetaxel decreased the gelatinase enzyme activity significantly in liver tissues compared to the control. This decline was also significant in the coadministration group compared to docetaxel administration alone in liver tissues (Fig. 4).

### Evaluation of total GST activity

**Fig. 5:**
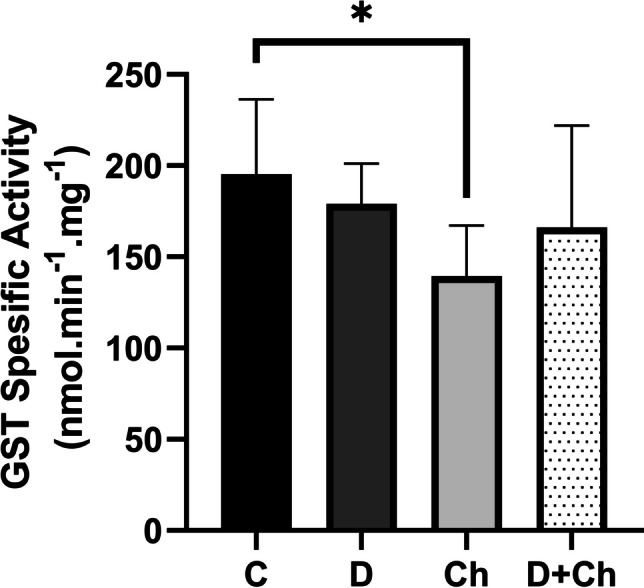
Liver GST activities. Each value is *mean* ± *SD* and * *p* < 0.05. GST, glutathione S transferase. C, control; D, docetaxel; Ch, chlorophyllin. *Mean* ± *SD*. *n* = 6

The results indicated that while docetaxel did not affect GST activity, chlorophyllin administration alone inhibited the GST activity significantly in tissues compared to the control. The coadministration of docetaxel and chlorophyllin also decreased the GST activity but was not statistically significant (Fig. 5).

### Evaluation of total GSH levels

**Fig. 6:**
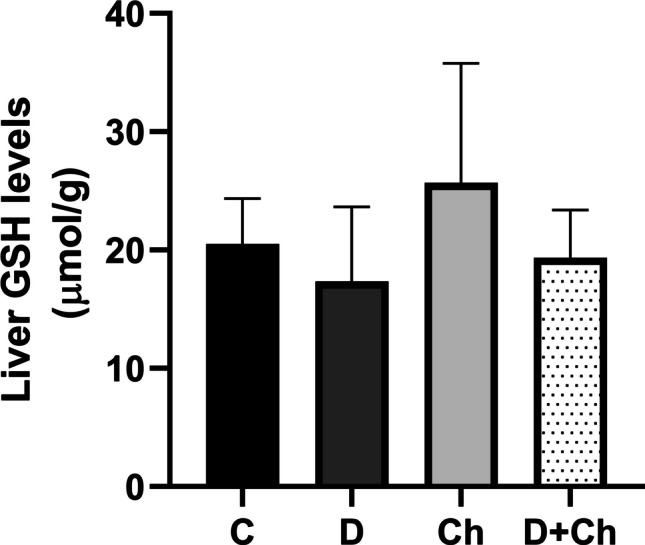
Liver total GSH levels. Each value is *mean* ± *SD*. GSH, glutathione; C, control; D, docetaxel; Ch, chlorophyllin. *Mean* ± *SD*. *n* = 3

The results indicated that GSH levels decreased with docetaxel administration, while increased with chlorophyllin administration in liver tissues, but the changes were statistically insignificant (Fig. 6).

### Evaluation of micrometastases by liver sectioning

**Fig. 7:**
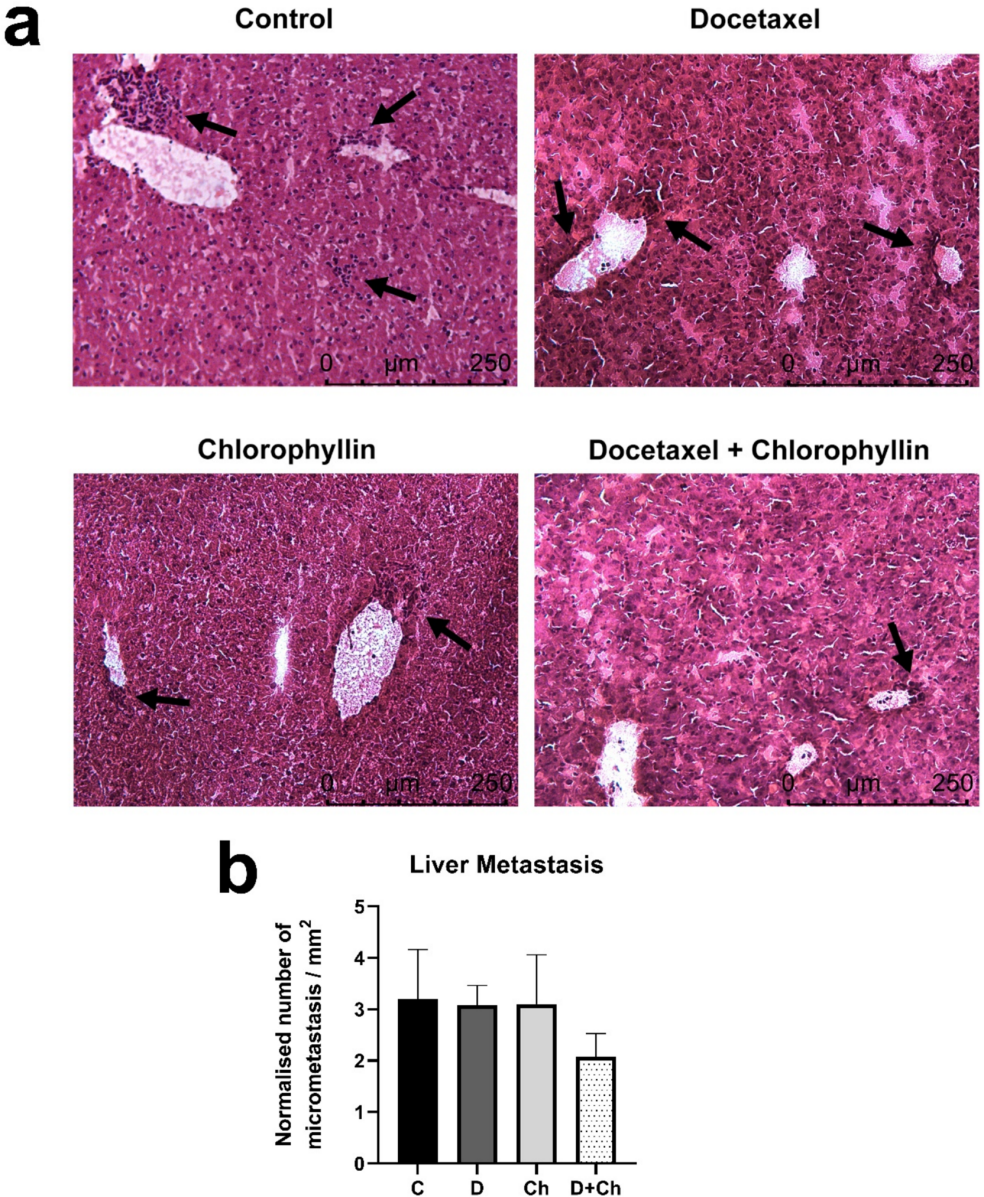
H&E sections of liver metastasis (**a**) and quantification of micrometastases (**b**). Scale bar: 250 μm. *Mean* ± *SD*. C, control; D, docetaxel; Ch, chlorophyllin

Liver tissues were sectioned and stained with H&E to examine the distant tissue metastasis (Fig. 7a). Results demonstrated that docetaxel or chlorophyllin administration alone did not affect the number of micrometastases; however, the coadministration of chlorophyllin and docetaxel decreased the micrometastases in the liver compared to the control group, but the decrease was statistically insignificant (Fig. 7b).

## Discussion

Triple-negative (ER, PR, and HER2 negative) breast cancer, having no receptor sensitivity, is the most lethal subtype of breast cancer because of its heterogeneity, aggressiveness, and lack of treatment options. Since it does not respond to hormonal therapy or HER2-targeted therapies, chemotherapy remains one of the main treatment methods (Won and Spruck 2020). However, drug resistance against chemotherapeutic agents is a major issue reducing the success of treatment, as well as the high incidence of metastases causing poor prognosis and, most importantly, mortality (Nedeljkovic and Damjanovic 2019). Therefore, novel therapy approaches and agents are critical in the treatment of triple-negative breast cancers and metastasis.

In this study, the antimetastatic activity of chlorophyllin is investigated in a triple-negative breast cancer animal model, and its related mechanisms are evaluated in vivo/in vitro. What makes the chlorophyllin compound interesting is that it is an antioxidant compound and has significant clinical translation potential because of its well-documented safety and tolerability. Clinical studies have used different doses. A phase I trial on aflatoxin-DNA adducts found 300 mg/day over 4 months to be safe and well-tolerated (Egner et al. 2001). Phase II studies, including those on radiation-induced hemorrhagic cystitis, further investigate the safety of sodium copper chlorophyllin at a daily dose of 750 mg (Krishnatry et al. 2024). With its favorable profile and low incidence of side effects, chlorophyllin holds promise for integration into cancer therapy, though further clinical trials are needed to establish its long-term safety and efficacy, especially in combination with standard cancer treatments. Furthermore, chlorophyllin participates in the inhibition of the GSTP1 enzyme, which has an increased expression level in tumor cells leading to drug resistance (Ozcan et al. 2018, Roomi et al. 2018, Dong et al. 2019, Ozcan et al. 2021). For this purpose, the effects of both a single administration of chlorophyllin and its coadministration with docetaxel, a taxane group anticancer drug, on triple-negative breast cancer metastasis were investigated.

Firstly, the MTT test was used to examine the effects of chlorophyllin or docetaxel on 4T1 cell viability, and IC_50_ values were determined. The IC_50_ values of docetaxel for 24- and 48-h incubation were found to be 31.45 and 34.4 µM, respectively, while the IC_50_ values of chlorophyllin were found to be quite high (148.6 µM and 115.5 µM). In the literature, similarly, chlorophyllin has been tested in the HT-1080 cells, a highly metastatic fibrosarcoma cell line, and shown that it does not affect cell viability or proliferation up to 50 µM (Roomi et al. 2018). In this study, the chlorophyllin dose was selected as 50 µM, accordingly.

Considering the increased IC_50_ value of docetaxel in 48 h, there may be several reasons to change cellular sensitivity to docetaxel. In one study, prostate cancer cell lines were treated with docetaxel for 24 h, and then the collected cells showed significantly higher proliferation rates at low docetaxel concentrations compared to normal cells (Sharma, Cwiklinski et al. 2023). Moreover, a study has shown that GSTP1 gene expression may increase in resistant cells following docetaxel treatment (Park et al. 2002). This further supports why a higher drug concentration is needed at 48 h to achieve the same cytotoxic effect, considering 4T1 cells’ short doubling time of 13.6 h (Simoes et al. 2015). GSTP1 is probably an additional but significant factor contributing to this resistance (Park et al. 2002). On the other hand, chlorophyllin, an antioxidant, seems to exhibit a different mode of action, with a relatively low cytotoxicity. The decreased IC_50_ of chlorophyllin at 48 h implies that the cells became more sensitive to the compound over time. One explanation is that while antioxidants can initially protect cells, prolonged exposure might deplete intracellular antioxidant reserves and make cells more susceptible to damage. In a study, it is shown that chlorophyllin inhibits the cellular thioredoxin reductase activity in a time-dependent manner in several cancer cell lines, induces ROS accumulation, and leads to cell death, with a lower IC_50_ value at 48 than 24 h (Sun et al. 2021). These results highlight the complexity of cellular responses to chemotherapeutic agents and antioxidants.

After determining the administration doses, the effects of chlorophyllin and docetaxel, both individually and in combination, on the 4T1 cell cycle were assessed using flow cytometry. At 24 h of incubation, the G0/G1 phase percentage in the control group was lower than in the chlorophyllin-treated and chlorophyllin + docetaxel co-treated groups, though the difference was not statistically significant. The observed higher G0/G1 phase in the treatment groups containing chlorophyllin compared to the control group indicates that chlorophyllin may possess an antiproliferative effect in the 4T1 cell line. In the literature, it has been shown that chlorophyllin increases the G0/G1 phase while decreasing the G2/M phase and has an antiproliferative effect in the MCF-7 breast cancer cell line (Chiu et al. 2005). In another study, chlorophyllin treatment has been reported to cause cell cycle arrest in HCT116 colon cancer cells through the increased expression of E2F1 and E2F4 transcription factors inhibiting the G1/S checkpoint step, which is an important cell cycle checkpoint (Chimploy et al. 2009).

The migration of cancer cells is the critical initial step of metastasis, enabling cancer cells to invade surrounding tissue and the local vasculature with increased cell motility (Yamaguchi et al. 2005). In this study, it was shown that the motility index of all treatment groups was significantly lower compared to the control group. Although both docetaxel and chlorophyllin could inhibit cell migration alone, their combined treatment had a substantially stronger effect. There was no available study in literature investigating the effects of combined use of chlorophyllin with anticancer drugs on cell migration; however, these results were in line with other studies reporting that certain antioxidants and/or phytochemicals administration, such as chlorophyllin, reduced the cell migration in aggressive cancer cell lines (Lirdprapamongkol et al. 2005, Roomi et al. 2018, Dehghani, Kooshafar et al. 2019). In a study on HT-1080 fibrosarcoma cells, it was shown that chlorophyllin dose-dependently reduced the cell migration, and this effect was highest at 50-µM chlorophyllin administration (Roomi et al. 2018).

MMP enzymes are involved in the degradation of the extracellular matrix and basal membrane. Several studies reported that MMP-2 and MMP-9, also known as gelatinase, were highly expressed in breast cancers (Cao et al. 2008, Li et al. 2017, Huang 2018). In parallel, many studies have been focusing on the research of MMP inhibitors to control metastatic processes (Winer et al. 2018). MMPs are first synthesized in zymogen (inactive pro-enzyme) form and their activation occurs through proteolysis or various agents (Rosenblum et al. 2007). In this study, gelatinase activity was investigated in both cell and tissue lysates, and the gelatinase activity was decreased in all the treatment groups. However, the only statistically significant decrease was seen in the chlorophyllin-contained groups in tissue. Although studies are showing the effect of chlorophyllin on MMP expression in the literature, there is no data about its possible inhibition mechanisms on the MMP enzyme activity (Nagini et al. 2015, Roomi et al. 2018). However, it is known that oxidizing agents such as reactive oxygen species (ROS) can participate in the activation of MMPs by breaking disulfide bonds (Fu et al. 2004, Kar et al. 2010). Therefore, the antioxidant property of chlorophyllin may lead to a decrease in MMP activity preventing the degradation of disulfide bonds. As a result, this study suggested that chlorophyllin might show anti-invasive and anti-metastatic effects as it had a direct inhibitory effect on the enzymatic activity of gelatinases. On the other hand, it is thought that the different findings observed between in vivo and in vitro may be due to the complexity of the tumor microenvironment (TME). The in vitro 4T1 model is deprived of major components of the TME, including M2 macrophages and cancer-associated fibroblasts (CAFs), which release factors that increase MMP secretion and alter the invasive characteristics of cancer cells (Chen et al. 2017, Rastegar-Pouyani et al. 2023). On the other hand, the in vivo liver metastatic niche represents a more physiological environment in which immune and stromal cells engage with tumor cells, likely enhancing the impact of the combination treatment of chlorophyllin and docetaxel. Although the potential contribution of the TME is considered in mediating the observed in vivo effects, further research is needed to address the complex mechanisms altering these treatment responses.

It has been reported that the GST enzymes are overexpressed in various cancer cell lines including breast cancer (Keith et al. 1990, Tew 1994). Chlorophyllin was shown to be a specific inhibitor of GSTP1. In a previous study of our group, it was demonstrated that chlorophyllin had a suppressing effect on the spread of breast cancer in an animal model chemically induced with N-methyl nitrosourea (Ozcan et al. 2018). In this study, a decrease in total GST activity in the liver tissues was observed only in the chlorophyllin group. However, our previous study demonstrated no significant difference in total GST activity in the chlorophyllin group (Ozcan et al. 2019). Even though total GST activity in the liver was measured, we specifically inhibited GSTP1 isoform, not the broader GST enzyme family. Despite GSTP1 inhibition, the cause of insignificance in overall GST activity may be due to other GST isoforms remaining uninhibited, and compensatory mechanisms may upregulate other GST isoforms, indicating the need for further investigations. On the other hand, chlorophyllin was effective in increasing glutathione levels in liver tissue, due to its antioxidant properties in addition to its GSTP1 inhibitory roles. It was considered that an increase in antioxidant glutathione levels indirectly suppresses MMP activity as the ROS could participate in the activation of MMPs (Fu et al. 2004, Kar et al. 2010). As a result, considering the antioxidant properties of chlorophyllin and glutathione, it could be possible to observe a decrease in MMP activation through the prevention of disulfide bond degradation.

Following the invasion of surrounding tissues cancer cells can extravasate to the distant tissue parenchymas through the vascular system and form small nodules named micrometastases and further macroscopic tumors by colonization that secondary tissue (Hanahan and Weinberg 2011). In this study, following the administration of docetaxel, chlorophyllin, and the combination of both; micrometastatic lesions (> 4 cells) were evaluated in liver sections (Madsen et al. 2015). In a single administration of docetaxel or chlorophyllin, there was no difference in the number of normalized micrometastases in the liver sections compared to the control; however, a decrease was observed following the coadministration of chlorophyllin with docetaxel. This data suggests that the coadministration of chlorophyllin and docetaxel may play a role in reducing the number of micrometastatic lesions. However, studies with larger sample groups are needed to observe a statistically significant difference.

One of the limitations of this study is the use of a single TNBC cell line. While the 4T1 model is a frequently used and well-established model to study TNBC, the results may not completely represent all TNBC subtypes. Including additional TNBC models in future research would further validate the findings and confirm the generalizability of combination therapy with chlorophyllin and anticancer drugs. In addition, the examination of different dose combinations of docetaxel and chlorophyllin for viability assessment was not included, which could provide valuable insights. Dose optimization in combined treatments should be investigated in future research to improve therapeutic potential.

## Conclusion

In conclusion, our results suggest that the coadministration of chlorophyllin and docetaxel may have a potential role in the control of metastatic processes in triple-negative breast cancer by suppressing cell migration and invasion which is mainly characterized by gelatinase enzyme activity. Therefore, chlorophyllin, a GSTP1 inhibitor, seems an interesting compound due to its safety and role in metastasis-related processes. Further and comprehensive studies are needed to be carried out to determine the exact inhibition mechanisms of chlorophyllin and to evaluate its possible positive role in metastatic cancer therapy alone or in combination with other anticancer drugs.

## Data Availability

The datasets generated during and/or analyzed during the current study are available from the corresponding author upon reasonable request.
